# Atomic Force Microscopy (AFM) As a Surface Mapping Tool in Microorganisms Resistant Toward Antimicrobials: A Mini-Review

**DOI:** 10.3389/fphar.2020.517165

**Published:** 2020-10-02

**Authors:** Zuzanna Grzeszczuk, Antoinette Rosillo, Óisín Owens, Sourav Bhattacharjee

**Affiliations:** ^1^ School of Physics, Technological University Dublin, Dublin, Ireland; ^2^ School of Veterinary Medicine, University College Dublin (UCD), Dublin, Ireland

**Keywords:** antimicrobial resistance, multidrug resistance, atomic force microscopy (AFM), nanomechanics, nanoindentation, single-cell force spectroscopy (SCFS), single-molecule force spectroscopy (SMFS)

## Abstract

The worldwide emergence of antimicrobial resistance (AMR) in pathogenic microorganisms, including bacteria and viruses due to a plethora of reasons, such as genetic mutation and indiscriminate use of antimicrobials, is a major challenge faced by the healthcare sector today. One of the issues at hand is to effectively screen and isolate resistant strains from sensitive ones. Utilizing the distinct nanomechanical properties (e.g., elasticity, intracellular turgor pressure, and Young’s modulus) of microbes can be an intriguing way to achieve this; while atomic force microscopy (AFM), with or without modification of the tips, presents an effective way to investigate such biophysical properties of microbial surfaces or an entire microbial cell. Additionally, advanced AFM instruments, apart from being compatible with aqueous environments—as often is the case for biological samples—can measure the adhesive forces acting between AFM tips/cantilevers (conjugated to bacterium/virion, substrates, and molecules) and target cells/surfaces to develop informative force-distance curves. Moreover, such force spectroscopies provide an idea of the nature of intercellular interactions (e.g., receptor-ligand) or propensity of microbes to aggregate into densely packed layers, that is, the formation of *biofilms*—a property of resistant strains (e.g., *Staphylococcus aureus, Pseudomonas aeruginosa*). This mini-review will revisit the use of single-cell force spectroscopy (SCFS) and single-molecule force spectroscopy (SMFS) that are emerging as powerful additions to the arsenal of researchers in the struggle against resistant microbes, identify their strengths and weakness and, finally, prioritize some future directions for research.

## Introduction

The emergence of widespread antimicrobial resistance (AMR) exhibited now by many commonly encountered pathogens including bacteria (e.g., Gram-positive: *Staphylococcus aureus*, *Streptococcus pyogenes*, *Mycobacterium tuberculosis*, and *Clostridium difficile*; and Gram-negative: *Escherichia coli*, *Klebsiella pneumoniae*, *Salmonella typhi*, *Pseudomonas aeruginosa*, and *Neisseria gonorroheae*), viruses (e.g., hepatitis B and C, herpes, and influenza), and fungi (e.g., *Candida albicans*, *Aspergillus fumigatus*, and *Cryptococcus neoformans*) against a range of popular antimicrobials, such as β-lactam antibiotics, macrolides, tetracyclines, aminoglycosides, fluoroquinolones, antihelminthics, and antifungals (Singer et al., 2016; Naylor et al., 2018; Hofer, 2019; Laws et al., 2019), in both human and veterinary medicine, such as the resistance noted against ivermectin in animal husbandry (O’Shaughnessy et al., 2019), is a major challenge today. Indiscriminate use of antibiotics due to their widespread availability and over-the-counter sales, often without prescription and in conjunction with poor sanitation, inadequate water purification, and wastewater management, as often occurs in developing countries, is posited to be a prime contributing factor to the surge of multidrug-resistant (MDR) strains, including methicillin-resistant *Staphylococcus aureus* (MRSA, Okwu et al., 2019) and vancomycin-resistant *Enterococcus* (VRE; Cetinkaya et al., 2000)—collectively termed as the *superbugs* (Davies and Davies, 2010; Khan and Khan, 2016). However, mutations within the genomes of microorganisms as an inherent trait to survive against antimicrobials are also a factor. Such AMR not only increases the mortality and morbidity from infectious diseases, but also increases drug toxicity in patients due to higher doses of antimicrobials required (Llor and Bjerrum, 2014). Additionally, AMR increases healthcare costs and creates a financial burden for resource-deprived countries. AMR is now declared a pandemic by the World Health Organization (WHO; MacIntyre and Bui, 2017) and one of the major healthcare challenges of this century, leaving the global population vulnerable to infectious diseases (**Supplementary material**).

A detailed discussion of the mechanisms of drug resistance falls beyond the scope of this review, but see Tenover, 2006; Munita and Arias, 2016; Peterson and Kaur, 2018. Most of the reported studies are based on bacterial resistance (Nikaido, 2009; Richardson, 2017), although accounts of viral (Irwin et al., 2016), fungal (Wiederhold, 2017), and parasitic resistance (Pramanik et al., 2019) are gradually being published. Scrutiny of the available literature reveals that resistant strains differ from sensitive ones in a few attributes, including biomechanical properties dictated by genetic makeup (Aguayo et al., 2015). It has been established that particularly in resistant bacterial strains, the cell walls are more rigid with reduced permeability and increased adhesiveness due to their altered composition (Harbottle et al., 2006; Aguayo et al., 2015), such as the cross-linked peptidoglycan and teichoic acid in bacteria (Muszanska et al., 2012); glycoproteins and phospholipids in virions (Ivanova et al., 2015); and chitins or glucans in fungi (Cowen et al., 2015). Such unusual and altered surface properties enable resistant strains to behave differently under biological and therapeutic circumstances. For example, the cell walls of resistant bacterial strains in general elicit greater stiffness and thickness (Tajkarimi et al., 2016) that deter the intracellular traffic of antimicrobial molecules, resulting in reduced drug efficacy. Similarly, due to increased adhesiveness, resistant bacteria are known to aggregate in densely packed layer(s) on biomaterials and produce *biofilms* due to both non-specific (e.g., acid-base and van der Waals) and specific (receptor-mediated binding to ligands) interactions (King and Korolik, 2017; Senneby et al., 2017; Carniello et al., 2018; Petridis et al., 2018), as reported for *Staphylococcus aureus/epidermidis*, *Pseudomonas aeruginosa*, *Porphyromonas gingivalis*, *Treponema denticula*, and *Tanerella forsythia*—the causative agents of infections refractory to antimicrobial therapy in cystic fibrosis (CF), endocarditis, osteomyelitis, sinusitis, otitis media, and nosocomial infections. An in-depth understanding of the cellular nanomechanical properties, including quantification of adhesive forces with nanoscale resolution and detection of molecular fingerprints and surface topography, have been hypothesized to be a strategy for identifying and then isolating resistant strains (Baptista et al., 2018). However, meeting such unique demands in biological samples high in aqueous content requires a sophisticated, versatile, robust, and highly sensitive analytic platform. Within the current inventory of analytic tools, atomic force microscopy (AFM) has emerged to satisfy most of the requirements described above, and therefore, much research into the biomechanics of microbes has relied on the use of AFM (Amro et al., 2000; Longo et al., 2013; Kelley, 2017; Kohler et al., 2019). This mini-review provides a brief understanding of the technique of AFM and investigates its possible applications.

## Modification of AFM Cantilevers/Tips and Force Spectroscopy

**Figure 1:**
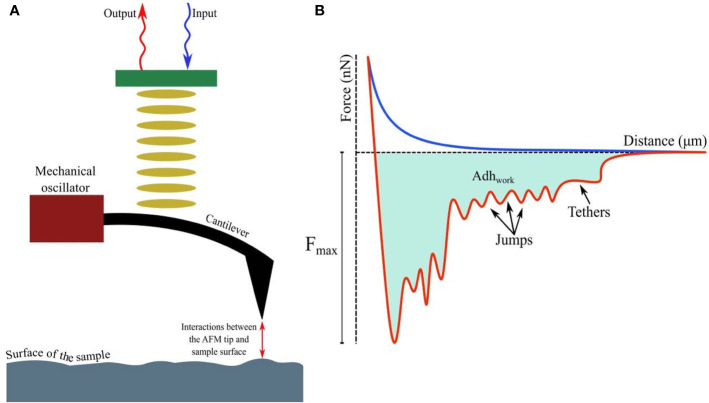
**(A)** Fundamental principles of AFM showing interactions between the tip and probed surface. **(B)** The force-distance curve while a (modified) tip is brought in proximity to another cell, bacterium, or biomaterial. The curves, when the tip is approaching or retreating from the sample, are drawn in blue and red respectively. Receptor-ligand bonds, when strained due to increasing detachment force, are marked as ‘jumps’, while ‘tethers’ appear when detachment is complete at F_max_. The shaded area denotes the total work done (Adh_work_) against adhesive forces.

A detailed account of the principles of AFM is out of the scope of this article; however, relevant literature is available for readers (Dufrêne, 2003; Liu and Wang, 2010; Lilledahl and Stokke, 2015). Attaining nanoscale resolution in surface topography and compatibility with aqueous conditions underlie the utility of AFM in medical microbiology, particularly as emerging data continue to suggest that in comparison to drug sensitive strains, resistant strains possess distinct surface and nanomechanical properties, such as elasticity and intracellular turgor pressure (Dorobantu and Gray, 2010). Additionally, by taking the spring constant (*k*) and vertical deflection (*d*) of the cantilever into account, force (*F*), such as the adhesive force (in nN or pN magnitude, Florin et al., 1994) acting between the sample and AFM tip (usually made of silicon nitride/Si_3_N_4_), can be quantified by Hooke’s law: F=*kd* and thus, a force-distance curve (Gavara, 2017; **Figure 1A**) can be developed revealing crucial information, such as quantification of (maximal) adhesive force and the nature of binding mediated by receptor-ligand interactions or formation of H-bonds (Zlatanova et al., 2000; Newton et al., 2017; **Figure 1B**) while the tip is approaching or retreating from the sample. Moreover, the process of modifying AFM tips with substrates (e.g., bacteria), molecules (carbon nanotube, peptide, alkene, thiol, and silanol) or particles (nanoparticles, glass, or latex beads), has led to considerable advancement in recent years (Gan, 2007; Barattin and Voyer, 2008; Wilson and Macpherson, 2009), resulting in unprecedented resolution (<1 Å). With such advantages, research into single-cell force spectroscopy (SCFS) or single-molecule force spectroscopy (SMFS) has increased (Helenius et al., 2008; Neuman and Nagy, 2008; Hoffmann and Dougan, 2012; Taubenberger et al., 2013). Furthermore, AFM tips can be used to cause indentation on microbial surfaces (*nanoindentation*), to extract information about the mechanical properties and chemistry of surfaces, or microbial cells (Chen, 2014; Kontomaris et al., 2019). To facilitate immobilization of the tip or samples on surfaces (e.g., glass, mica, gold or silica) and minimize detachment (Suo et al., 2008; Allison et al., 2011) while conducting measurements, various adhesive materials have been used, such as poly-L-lysine, glutaraldehyde, polyethyleneimine, gelatin, and the biocompatible polymer polydopamine/poly-DOPA (Lee et al., 2007; Dreyer et al., 2012; Li et al., 2016; Li et al., 2017), in addition to simple surface adsorption.

## AFM-Based Microbial Studies

**Figure 2:**
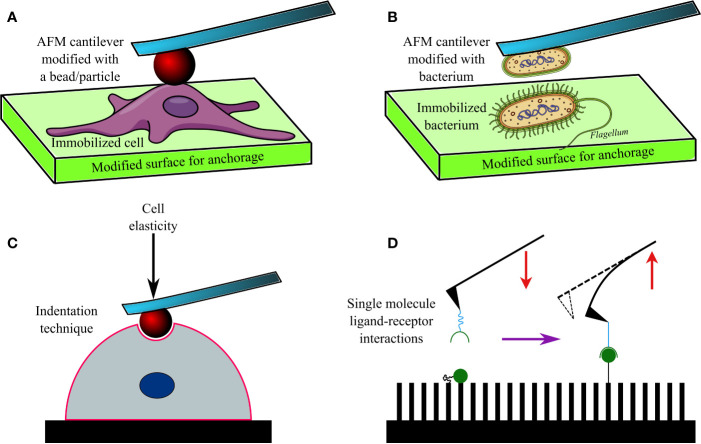
A diagram showing an AFM cantilever modified with a bead **(A)**, and bacterium **(B)** to perform indentation studies **(C)** on an immobilized cell or bacterium. Modified AFM tips are also used to investigate various ligand-receptor interactions **(D)** on functionalized surfaces.

The AFM cantilevers/tips have often been modified/tethered by attaching a bacterium with the help of adhesive materials (**Figure 2**). Cantilevers coated in poly-DOPA and attached to *Escherichia coli* bacterium have been used to probe biofilms of various microorganisms, including *Massilia timonae*, *Pseudomonas aeruginosa*, and *Bacillus subtilis* (Harimawan et al., 2011). However, the gradual heating of the cantilever upon being exposed to a laser beam affects cellular viability (Beaussart et al., 2013a), while the lack of uniformity during contact remains a challenge in SCFS. Hence, tip-less cantilevers attached to glass or latex beads (300 nm–1 µm) are being increasingly used. Functionalized cantilevers have also been used to study the adhesive force between bacterial cells and hard surfaces, which increases over time of contact (usually <60 s), for example, between *Staphylococcus epidermidis* and fibrinogen-coated surfaces (Herman et al., 2013); *Lactobacillus rhamnosus* GG and mucin epithelial cells or hydrophobic surfaces (Sullan et al., 2014); *Staphylococcus carnosus* and hydrophilic/hydrophobic silicon wafers (Loskill et al., 2012); *Escherichia coli* and corundum (Al_2_O_3_) or hematite (Fe_2_O_3_) nanoparticles (Zhang et al., 2011); oral *Streptococci* and saliva-coated tooth enamel (Mei et al., 2009); oral microbiome and human saliva-coated bovine tooth enamel (Wessel et al., 2014); *Staphylococcus aureus, Pseudomonas aeruginosa* or *Serratia marcescens* and contact lenses coated in polypropylene and silver (Qu et al., 2013a), or brush-coated silica nanoparticles (Qu et al., 2013b).

AFM has been used to investigate the adhesion of bacteria to other microbes, cells, and molecules. This technique has found its niche, particularly in investigating the dynamics and nature of interactions between microbial surface receptors and ligands (Hinterdorfer and Dufrêne, 2006; **Figure 2B**). For example, gold AFM cantilevers coated in vancomycin (~1 nm thickness) have been used to determine the surface density of D-Ala-D-Ala terminals of peptidoglycans expressed on *Lactococcus lactis* (Gilbert et al., 2007). Similarly, polyethyleneimine-coated cantilevers functionalized with *Lactococcus lactis* bacterium have been used to measure short (100–200 nm) and long (600–800 nm) distance interactions between the bacterium and porcine gastric mucin (Le et al., 2013). Such short and long-distance interactions were determined by elongated *pili* and mucin-binding proteins of the bacterium, respectively. In another study, cantilevers attached to a glass bead and coated in poly-DOPA were bioconjugated to *Staphylococcus aureus*, and SCFS was conducted (Beaussart et al., 2013b) to investigate its interactions with *Candida albicans*, a fungus often co-isolated from biofilms of *Staphylococcus aureus* (Ovchinnikova et al., 2012) in nosocomial infections (e.g., infected catheters/tubes), intra-abdominal sepsis and deep-seated abscess. The obtained data 4revealed that the peptides and lectin receptors on bacterial surface and Als-proteins plus *O*-mannosylated sites on fungi were major drivers of such interactions. Moreover, *Staphylococcus aureus* was noted to possess a higher affinity toward yeast tubes than the yeast cells of *Candida albicans*. The role of LapA *adhesin* protein secreted by *Pseudomonas fluorescens* to enhance binding on hydrophobic surfaces was confirmed by assessing interactions between an AFM tip tethered with anti-hemagglutinin (HA) antibody and HA-tagged LapA deposited on hydrophobic alkanethiol-coated surfaces upon bacterial colonization (El-Kirat-Chatel et al., 2014a; **Figure 2D**). In a further study the surface density of LapA adhesins on *Pseudomonas fluorescens* was shown to be ~450 sites/µm^2^ and the adhesive force between surfaces and bacteria was shown to be increased in highly adhesive LapA+ mutant strains (El-Kirat-Chatel et al., 2014b).

Interestingly, AFM has been frequently used to determine hardness and elasticity of cell surfaces by inflicting *nanoindentation* with the tips, while Young’s moduli of cells were calculated from cantilever deflection and its movement in the *z*-direction (Webb et al., 2011; **Figure 2C**). Nanoindentation studies can be performed under different conditions, including aqueous ones, and provide in-depth information on nanomechanical properties of cells. Such a study conducted on *Escherichia coli* revealed heterogeneous stiffness of bacterial cells, with stiffer areas indicating proximity to intracellular organelles (Longo et al., 2012). Nanoindentation studies conducted on seven bacterial strains (*Comamonas testosterone*, *Aeromonas punctata*, *Raoultella ornithinolytica*, *Bacillus cereus*, *Shewanella putrefaciens*, *Shewanella oneidensis*, and *Desulfovibrio vulgaris*) established a correlation between their nanomechanical properties and the ability of the bacteria to aggregate (Wang et al., 2012). Nanomechanical investigations based on such indentation techniques have also helped to understand the effects of antibacterials such as ticarcillin and tobramycin (Formosa et al., 2012a), or novel antibiotics (Formosa et al., 2012b) on *Pseudomonas aeruginosa*; alginate oligosaccharide (*OligoG*) of low molecular weight on *Acinetobacter baumannii* and *Pseudomonas aeruginosa* biofilms (Powell et al., 2013) and antimycobacterial drugs (ethambutol and isoniazid) on *Mycobacterium* sp. strain *JLS* (Wu and Zhou, 2009). By using tip-less cantilevers functionalized with *Staphylococcus aureus/epidermidis*, *Streptococcus salivarius* bacteria and a maximal loading force of 3 nN, it was discovered that Gram-positive bacteria demonstrated heterogeneous elasticity, comprising a rigid core and deformable cylindrical surface contact areas (Chen et al., 2012). Interestingly, *Salmonella typhimurium* regained its normal morphology and ability to divide after repeated punctures by AFM tips at multiple locations (Suo et al., 2009). Only a few researchers have reported success in imaging and mapping the distribution of proteins and protein complexes on bacterial cell walls of *Halobacterium halobium* (Worcester et al., 1988; Butt et al., 1990) and *Deinococcus radiodurans* (Karrasch et al., 1994) with lateral and vertical resolutions of 1 nm and 0.1 nm respectively. Furthermore, the effect of ambient factors, such as pH, temperature, and ionic strength, on surface proteins of bacteria has been investigated by SCFS in *Halobacterium salinarium* (Oesterhelt et al., 2000). Similarly, the spatial distribution of polysaccharides on the surface of *Lactobacillus rhamnosus* GG, both wild type and CMPG5413 mutant with reduced production of polysaccharides, was probed with unmodified and modified AFM tips attached to lectin and concanavalin A (Francius et al., 2008). In another study, the stacks of lipopolysaccharide (LPS) molecules on *Escherichia coli* were imaged by AFM with a lateral and vertical resolution of 50 Å and 5 Å, respectively (Amro et al., 2000). The role of LPS on adhesion of *Escherichia coli* was later also confirmed by force spectroscopy (Abu-Lail and Camesano, 2003). In a follow-up study on eight *Escherichia coli* strains, the length of LPSs on virulent strains carrying O-antigens was reported to vary between 17 ± 10 nm to 37 ± 9 nm; whereas they were much shorter (3 ± 2 nm to 5 ± 3 nm) in strains lacking the O-antigen (Strauss et al., 2009). AFM has also been used to image bacterial appendages, such as pili and flagella, as well as their capsules (Tollersrud et al., 2001; Touhami et al., 2006; Dorobantu et al., 2008; Stukalov et al., 2008).

AFM has been used to measure intracellular bacterial turgor pressure that is important for maintaining cellular morphology and function (Beveridge, 1988; Doyle and Marquis, 1994; Walsby et al., 1995). It is usually higher in Gram-positive (20–50 atm) than in Gram-negative (3–5 atm) bacteria. Such AFM-based measurements of intra-bacterial turgor pressure have been conducted in Gram-negative *Magnetospirillum gryphisw* (85–150 kPa in a buffer; Arnoldi et al., 2000), Gram-positive *Enterococcus hirae* (400–600 kPa in water) and Gram-negative *Pseudomonas aeruginosa* (10–20 kPa in a growth medium and 150–400 kPa in water; Yao et al., 2002). Young’s (elastic) moduli for various strains were measured by AFM-based indentation studies and were found to be lower (1–10 kPa) than those of biomolecules such as proteins (0.5 GPa; Kuznetsova et al., 2007). Viable cells, however, demonstrated lower Young’s moduli (3.0 ± 0.6 MPa) than those with compromised cell walls (6.1 ± 1.5 MPa) or dead cells (Cerf et al., 2009). Such studies also revealed that in Gram-negative *Shewanella putrefaciens*, an increase in pH of the suspension medium from 4 to 10 resulted in a thicker cell wall with reduced stiffness (Gaboriaud et al., 2005).

## Challenges

It is difficult to model the interactive forces between a bacterium-probe attached to a cantilever and cell probes like bacterium/cell/ligands, because the current mainstay of modeling such interactions, the Derjaguin-Landau-Verwey-Overbeek (DLVO) theory, assumes that interacting surfaces are perfectly smooth and non-functionalized (Attard, 2003; Dorobantu et al., 2009). However, this is not the case in SCFS/SMFS studies, in which microbial surfaces are rough and often decorated with a diverse set of molecules, including biopolymers and macromolecules (Feick et al., 2004; Gotzinger and Peukert, 2004). A modified version of the DLVO model, such as the extended DLVO (XDLVO) that includes hydrophobic interactions or accounts for the polymers present on interacting surfaces, provides a more accurate model (Jucker et al., 1998), but further improvement is still necessary. Moreover, SCFS/SMFS techniques are time-consuming, logistically demanding and labor-intensive, making their development into user-friendly point-of-care diagnostics unlikely in the forseeable future. Therefore, it is difficult to obtain statistically relevant clinical datasets, especially when performing comparative studies on multiple bacterial strains. The use of basic AFM requires training, while high-end utilization, for example cryogenic AFM, also requires considerable technical expertise and robust background knowledge, which may be an obstacle for interdisciplinary researchers from disparate disciplines.

## Perspectives

Performing sophisticated SCFS/SMFS studies with modified AFM tips has opened novel avenues to investigate many unanswered questions regarding host-microbe or pathogen-surface interactions. A better understanding of the intricacies of such interactions is crucial for developing high efficacy antimicrobial therapeutics. However, current technological challenges need to be addressed to convert currently available techniques into more user-friendly and flexible ones. In comparison to the molecular techniques for detecting AMR, such as the polymerase chain reaction and DNA-microarray technology (Fluit et al., 2001; Tan, 2003), AFM presents a simpler tool with lesser sample preparation requirements and greater cost-effectiveness. Additionally, techniques like SCFS/SMFS enable topographical analyses, including measurement of stiffness and elastic moduli, of various surfaces of interest, such as biofilms. Recent advances in AFM instrumentation have enabled studies on various organs of the human body, such as the brain (Viji Babu and Radmacher, 2019), lungs (Sicard et al., 2018) and liver (Saneyasu et al., 2016), including various physicochemical attributes, such as stiffness of the extracellular matrix (Jorba et al., 2017) and tissue architecture (Zapotoczny et al., 2017). In the future, it will be interesting to see whether AFM can differentiate between sensitive and resistant microorganisms based on measurements conducted on slices of infected tissues. However, tip modification techniques need further improvement to ensure higher resolution imaging and ultrasensitive measurements on biological samples and the ability to establish uniform contact areas with defined 2D and 3D geometry. Furthermore, systematic studies of resistant strains need to be performed, which will be a challenge given that such strains are rarely available for laboratory research and when available, pose a serious health-and-safety risk; working with such resistant microorganisms within non-containment AFM labs is realistically impracticable. Future research should be focused on these important areas to develop a growing range of applications for nanotech-tools in clinical microbiology, including investigation of AMR microbes.

## Author Contributions

ZG, AR, and ÓO conducted the literature survey and wrote the draft. SB supervised the entire project. All authors contributed to the article and approved the submitted version.

## Funding

SB would like to thank UCD Research for funding.

## Conflict of Interest

The authors declare that the research was conducted in the absence of any commercial or financial relationships that could be construed as a potential conflict of interest.
