# Supplementary material for: Atomic Force Microscopy (AFM) As a Surface Mapping Tool in Microorganisms Resistant Toward Antimicrobials: A Mini-Review
Source: Front Pharmacol. 2020 Oct 2;11:517165. doi: 10.3389/fphar.2020.517165 (PMC7567160; doi:10.3389/fphar.2020.517165)
Supplement: Supplementary file 1 [file DataSheet_1.pdf]

Supplementary Material

The WHO Priority Pathogens List for the development of novel antibiotics.

Organism	Type	Route of exposure	Antibiotics	Resistance	Multi virulent?	Virulence	Priority
C. difficile	Bacillus + anaerobic	Nosocomial	Metronidazole, vancomycin	Low	Yes	Virulent	High
S. aureus	Coccus + anaerobes	Human contact	Methicillin-resistant, vancomycin intermediate and resistant	Medium	Yes	Dangerous	High
N. gonorrhoeae	Diplococci + aerobic	Sexually transmitted	3rd generation cephalosporin-resistant, fluoroquinolone-resistant	Medium	Yes	Worrying	High
M. tuberculosis	Bacillus not classified as aerobic or anaerobic	Air-borne	Rifampicin, Isoniazid	Medium	Yes	Virulent	High
A. baumannii	Coccobacillus + aerobic	Medical facilities/ nosocomial fomites	Carbapenem-resistant, tigecycline,	High	Yes	Worrying	Critical
E. coli	Bacillus + facultative anaerobe	Needle injury/ mucosal exposure of the eye/ aerosol inhalation	Carbapenem-resistant, 3rd generation cephalosporin-resistant	High	Yes	Worrying	Critical
K. pneumoniae	Bacillus + facultative anaerobe	Respiratory tract/ bloodstream	Carbapenem-resistant, 3rd generation cephalosporin-resistant	Medium	Yes	Worrying	Critical
P. aeruginosa	Bacillus + facultative anaerobe	nosocomial	Carbapenem-resistant	Medium	Yes	Worrying	Critical
